# Multidrug-resistant *Staphylococcus aureus* isolated from chicken nuggets sold at superstores in Mymensingh, Bangladesh

**DOI:** 10.5455/javar.2022.i629

**Published:** 2022-11-19

**Authors:** Qurra-Tul Ain, Mohammad Arif, Fateha Akther Ema, Most. Shumi Akhter Shathi, Md. Ariful Islam, Mst. Minara Khatun

**Affiliations:** Department of Microbiology and Hygiene, Faculty of Veterinary Science, Bangladesh Agricultural University, Mymensingh, Bangladesh

**Keywords:** Multidrug-resistant, *Staphylococcus aureus*, chicken nugget, antibiotic sensitivity, Mymensingh

## Abstract

**Objective:**

This study was conducted to determine the colony forming units (CFU) to isolate, identify, and antibiotic sensitivity of *Staphylococcus aureus *from chicken nuggets (CN).

**Materials and Methods:**

Sixty CN samples from two brands were collected from different superstores in Mymensingh, Bangladesh. Uncooked, oven-cooked (OC), or gas stove-cooked (GSC) CN samples were inoculated onto mannitol salt agar and blood agar.

**Results:**

The total staphylococcal count (TSC) for uncooked CN ranged from log 4.68 to log 5.11 CFU/gm. For OC CN, TSC ranged from log 3.29 to log 3.62 CFU/gm. For GSC CN, TSC ranged from log 3.09 to log 3.49 CFU/gm. Relative to uncooked CN, microwave oven-cooked and GSC samples significantly reduced the TSC of CN (*p* < 0.01). Using the polymerase chain reaction assay and standard biochemical testing, only 8 out of 60 CN samples contained *S. aureus.* *Staphylococcus aureus* were resistant to Ampicillin (100%), Amoxicillin (100%), Oxacillin (75%), Cefixime (87.5%), Doxycycline (75%), intermediately sensitive to Erythromycin (25%), Cephalexin (12.5%), Ciprofloxacin (25%), Gentamicin (12.5%), Doxycycline (12.5%) and sensitive to Oxacillin (25%), Azithromycin (100%), Erythromycin (75%) Cephalexin (87.5%), Cefixime (12.5%), Chloramphenicol (100%), Ciprofloxacin (75%), Gentamicin (87.5%), Doxycycline (12.5%), and Vancomycin (100%).

**Conclusion:**

This study reports the first isolation and identification of *S. aureus* from CN in Bangladesh. GSC CN was better than OC and uncooked CN. Data also suggest that CN is contaminated with multidrug-resistant *S. aureus*, which poses a public health hazard.

## Introduction

Antimicrobials are widely used in the livestock sector, where most of the time they are applied sub-therapeutically for growth promotion and routine disease prevention [1]. This practice contributes to the production and spread of multidrug-resistant (MDR) bacteria. Food that can be prepared and served very quickly or sold in a restaurant or store using preheated or precooked ingredients is known as fast food [2]. Bangladesh’s upper and middle classes are quickly finding that fast food is a tasty and easy way to get food. People who live in towns are more likely to consume fast food than those who live in rural areas. The busy and hectic life schedules of urban people encourage them to consume various fast foods in their diet [3] as they have less time to make food at home [2]. KFC, McDonald’s, Pizza Hut, Shawarma House, and Domino’s Pizza are well-known fast-food sellers of pizza, burgers, soups, fried chicken, chicken fingers, and chicken nuggets (CN), the most popular fast foods in Bangladesh.

CN are one of the most popular fast foods enjoyed by people of all ages due to their excellent nutritional content and delicious taste. A CN contains valuable nutritional ingredients like fat, protein, vitamins, and minerals. The CN is made of slurry chicken breasts that can be shaped and breaded or battered, and after mixing with egg white and bread crumbs, they are deep fried or baked. Unfortunately, *Staphylococcus aureus* can potentially exist and thrive in the CN due to the unfortunate short heat processing time (approximately 4 min). Furthermore, unsanitary preparation, handling, and packaging practices can easily contaminate CN [4,5]. Food contamination with microorganisms can shorten the shelf life of food and put people’s health at risk by exposing them to food-borne infections and poisonings [6,7]. It can also hurt the economy because food spoils.

Staphylococcal food poisoning (SFP) is happening too often around the world as a very common food-borne disease with the highest occurrence next to salmonellosis [8]. *Staphylococcus* aureus is an important food-borne pathogen that causes everything from minor skin infections to serious conditions like pneumonia and septicemia [9]. *Staphylococcus* aureus is mainly found on the skin, in sores, infected eyes, and in the nose, throat, saliva, and fecal matter of humans. Food contaminated with S. aureus or its toxins can cause food poisoning. The contamination may occur from faulty preservation methods, unhygienic handling practices, cross-contamination from food handling instruments, or people carrying S. aureus in their nares and on their skin [10,11]. SFP can cause many of the same symptoms as other foodborne illnesses, including nausea, abdominal cramps, vomiting, or diarrhea [12]. *Staphylococcus* aureus causes infections like bacteremia, pneumonia, myocarditis, osteomyelitis, acute endocarditis, pericarditis, encephalitis, meningitis, mastitis, and scalded skin syndrome [13].

Antimicrobial resistance (AMR) poses a significant risk to public health worldwide and has emerged as a substantial clinical practice and healthcare [14]. AMR is caused by the misuse and overuse of antibiotics, which has led to the evolution of antibiotic-resistant bacteria and genes in all types of animal farming environments in Bangladesh. These predisposing factors include insufficient veterinary healthcare, monitoring, and regulatory services; intervention from numerous informal animal health service providers; and farmers’ lack of knowledge regarding drugs. MDR bacteria with extreme resistance against antibiotics recommended for use in animals and humans have been reported to be a potential public health hazard in Bangladesh [15]. Many of the *S. aureus* isolates are found to be resistant to a variety of antibiotics, and due to the emergence of these multidrug-resistant bacterial strains, public health is facing a great significant issue. Methicillin-resistant *S. aureus* (MRSA) and vancomycin-resistant *S. aureus* (VRSA) are examples of common types of drug-resistant *Staphylococcus*. Humans can contract resistant bacteria from animals by consuming animal products like milk, meat, eggs, etc., through direct or close contact with them, or by being exposed to unsanitary environments [16]. Based on the information above, this study was done to estimate the total amount of *Staphylococcus* in CN, isolate and identify *S. aureus* from CN, and figure out how susceptible the isolated strains are to antibiotics.

## Materials and Methods

### Collection and processing of samples

For bacteriological analysis, 60 CN samples from brands, such as Brand 1 = 30 CN and Brand 2 = 30 CN were purchased from different superstores in Mymensingh, Bangladesh. CN samples were placed in an ice box and immediately transported to the laboratory of the Department of Microbiology and Hygiene, Bangladesh Agricultural University (BAU), Mymensingh, and evaluated within an h of collection. Before bacteriological analysis, CN samples were divided into three categories:

(i) Uncooked CN (*n = *20)—taken from the packet directly as it is produced.

(ii) Oven-cooked (OC) CN (*n = *20)—it was treated at 250°C for 3 min (800 w) in an oven at the central lab.

(iii) Gas stove-cooked (GSC) CN (*n = *20)—it was treated with oil at medium-high heat (230°C ) for 5–10 min.

All three types of samples were uniformly homogenized using sterile Phosphate-buffer solution (PBS) in a sterilized mortar and pestle as per the recommendation of ISO [17] by placing a 10-gm sample of each CN into 90 ml PBS. Later on using the vortex mixture machine (VM-2000, Digisystem Laboratory Instruments Inc, Taiwan), 10-fold serial dilutions followed ranging from 10^−2 ^to 10^−5^ and were cultured by the standard plating method of ISO [17].

### Enumeration of total Staphylococcal count (TSC)

About 0.2 ml from each 10-fold serial dilution was transferred and immediately spread on mannitol salt agar (MSA) and incubated at 37°C for 24 h. After incubation, plates containing 30–300 colonies were counted. The TSC was calculated according to standard protocol [17]. The number of colony forming units (CFU) per gram of CN samples was used to express the results of the TSC. 

### Isolation and identification of Staphylococcus spp.

Following Ceesbrough’s recommended procedure [18,19], bacteria were isolated and identified. Samples were inoculated into the nutrient broth and incubated at 37°C for 24 h. The overnight enriched culture was streaked in duplicate plates onto MSA and blood agar (BA). The inoculated plates were incubated at 37°C for 24 h. One colony was subcultured repeatedly until the pure culture was produced. The identification of bacteria was performed based on colony morphology such as size, shape, color, and texture, Gram’s staining reaction, hemolysis test, and biochemical tests like sugar fermentation test (dextrose, maltose, lactose, sucrose, and mannitol), methyl red (MR), Voges–Prauskauer (VP) tests, and catalase and coagulase tests as described by Cheesbrough [19]. 

### Confirmation of S. aureus by polymerase chain reaction (PCR)

By utilizing a boiling process to lyse the bacterium, template DNA was created [20]. The staphylococcal enterotoxin A (*sea*) gene, the staphylococcal *nuc* gene, and the *16S rRNA* gene of *Staphylococcus* spp. were all identified by PCR using three sets of primers (Table 1) to specifically identify *S. aureus* strains that produce enterotoxins. Genus-specific PCR reactions for the detection of *Staphylococcus* spp. were performed using a thermocycler with the following program: 1 cycle of initial denaturation at 94°C for 5 min, 35 cycles each consisting of denaturation with 94°C for 1 min, annealing at 68°C for 45 sec, extension at 72°C for 1 min, and a final extension step of 10 min at 72°C. On the other hand, molecular detection of enterotoxin-producing *S. aureus* was performed with 1 cycle of initial denaturation at 94°C for 5 min, 30 cycles each consisting of denaturation at 94°C for 2 min, annealing at 55°C for 2 min, extension at 72°C for 1 min, and a final extension step of 10 min at 72°C. In both cases, amplified PCR products were passed through electrophoresis in 1.5% agarose gel for 30 min at 100 volts, followed by staining with ethidium bromide and finally visualized under a UV trans-illuminator.

### Antibiotic sensitivity assay

Antibiotic sensitivity tests were conducted against all *Staphylococcus *spp. isolates to determine their pattern of bacterial resistance. According to the recommendations of the Clinical and Laboratory Standard Institute [24], all isolates were tested using the disc diffusion method against 12 different commonly used antibiotics (Amoxicillin, Ciprofloxacin, Erythromycin, Azithromycin, Chloramphenicol, Ampicillin, Gentamicin, Doxycycline, Cefixime, Cephalexin, Oxacillin, and Vancomycin). The diameter of the zone of inhibition was measured, and the results were compared with the values from the National Committee on Clinical and Laboratory Standards for interpretation. *Staphylococcus *isolates were then classified as resistant, intermediate, or sensitive against a particular antibiotic. Isolates that are non-susceptible to at least one antimicrobial agent in three or more antimicrobial classes were recorded as MDR phenotype [25].

### Statistical analysis of experimental data

The data of TSC of CN samples were analyzed for statistical significance using *p*-value and Duncan’s multiple range test. Data were analyzed by Statistical Package for the Social Sciences (SPSS) version 11.5 (SPSS Inc., Chicago, IL).

## Results and Discussion

### Total staphylococcal count of CN

*Staphylococcus aureus* is one of the leading etiologic agents of food poisoning causing human gastrointestinal illness [26]. *Staphylococcus* causes various pathological conditions like bacteremia, urinary system infections, systemic diseases, and osteomyelitis in humans and animals [27]. In most cases, bacterial food poisoning results from the toxin produced by *S. aureus* [28]. Most individuals and animals have *Staphylococcus* spp. on their skin and noses, which can contaminate food when handled by people who do not practice good hygiene. Bacterial growth is accompanied by the generation of toxins in food if *Staphylococcus* spp. contaminates the food. Although the pathogen is killed by cooking, the heat-stable toxins are not destroyed and will still be able to cause illness. Foods like sliced meats, pastries, puddings, fast food, and sandwiches become very risky for consumers if they get contaminated with *Staphylococcus* spp. because these foods are not cooked after handling.

**Figure 1.**
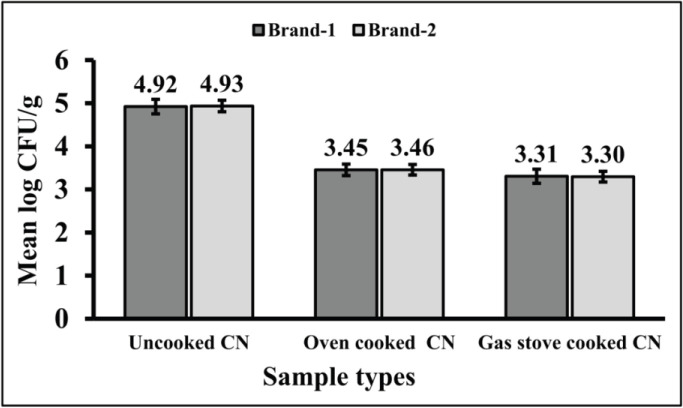
TSC of CN. Results are expressed as mean log CFU/gm. In uncooked CN, OC CN, and GSC CN, the mean log CFU/gm of TSC from brand-1 samples was 4.92, 3.45, and 3.31, respectively. Similarly, the TSC values from the brand-2 chicken nugget samples were 4.93, 3.46, and 3.30, respectively.

In this study, out of 60 CN samples, 8 were found positive for *S. aureus, *and 52 were found positive for other *Staphylococcus* spp. The TSC from two different places is presented in Figure 1.

**Table 1.** List of oligonucleotide primers used in this study.

Primer	Sequence (5’ to 3’)	Size (bp)	References
*Staphylococcus* sp. *16S*	F- GGAGGAAGGTGGGGATGACG	241	[21]
	R- ATGGTGTGACGGGCGGTGTG		
*S. aureus* *nuc*	F- GCGATTGATGGTGATACGGTD	279	[22]
	R- AGCCAAGCCTTGACGAACTAA AGC		
*Staphylococcus *sp. *sea*	F- TTGGAAACGGTTAAAACGAA	120	[23]
	R- GAACCTTCCCATCAAAAACA		

Samples were cultured on MSA to determine the TSC. The mean ± SD value (CFU ± SD/gm) of TSC from brand-1 samples were 4.92 ± 0.168, 3.45 ± 0.133, and 3.31 ± 0.163 in uncooked CN, OC CN, and GSC CN, respectively. Similarly, the TSC values were found as 4.93 ± 0.133, 3.46 ± 0.126, and 3.30 ± 0.123 respectively from the CN samples of brand-2. These results are nearly similar to the findings of Wimalasekara and Gunasena [29] who had found 10^4^ CFU/gm in meat-based fast-food samples in Colombo, Sri Lanka. Similarly, recent findings are in strong agreement with the research findings of total TSC from street-vended fast food in Bangladesh conducted by Hoque et al. [30], Adimasu et al. [31], and Sabuj et al. [32]. Akhi et al. [33] found 68% (17/25) of *S. aureus* from chicken meat samples.

From the findings of this study, it is clear that the highest Staphylococcal count was obtained from the uncooked CN samples of both brands which may lead to possible health hazards through the production of a significant amount of enterotoxin within a very short time [30]. The mean log CFU of GSC CN is lowest and OC CN is lower compared to uncooked CN which is statistically significant (*p* < 0.001), (significant at 1% level of probability, *p* < 0.01). Hence it can be concluded that heat treatment with a gas stove can decrease the Staphylococcal CFU/gm from the CN by 10-fold.

*Staphylococcus* spp. in CN samples suggests potential contamination from the food workers’ mouths, eyes, noses, or other skin-contact areas [32]. Tambekar et al. [34] stated that food contamination might be more likely if sellers have contaminated hands and lack knowledge of personal hygiene, hygiene procedures, and food safety.

### Identification of bacteria

Different tests were used to identify *Staphylococcus* spp. from the CN samples. The results of cultural characteristics, Gram stain, biochemical tests, and molecular identification by genus and staphylococcal *enterotoxin A* (*sea*) gene-specific PCR identified the microorganisms were similar to previous research findings [34,35].

### Hemolytic characteristics

On 5% of sheep BA, *S. aureus* produces β-hemolysis, similar to the findings of Jahan et al. [36]. Among 60 CN samples, only 8 exhibited β-hemolysis, and the remaining 52 samples grew as bright white colonies on BA without any hemolysis.

**Figure 2.**
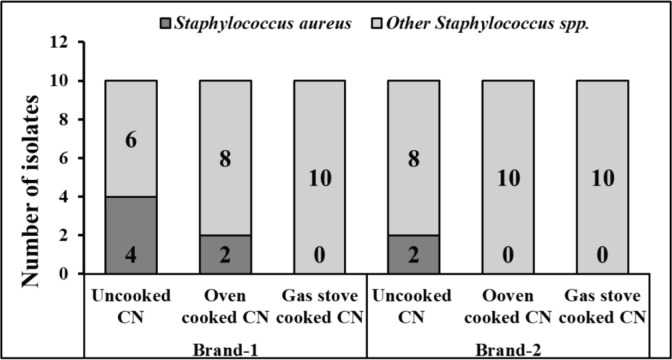
Summary of isolation of *S. aureus* and *Staphylococcus* spp. from two different brands of CN. Out of 60 CN samples, 8 tested positive for *S. aureus* and 52 tested positive for other Staphylococci. In the case of brand-1, 4 of the 10 uncooked CN samples were confirmed to be *S. aureus* positive. 2 of the 10 OC CN samples from brand-1 were revealed to be *S. aureus* positive. For brand 2, two out of ten uncooked CN samples were found positive for *S. aureus. *Legend: CN-Chicken nugget.

In this study, out of 60 CN samples, 8 were found positive for *S. aureus*, and 52 were found positive for other staphylococci. In the case of brand-1, out of 10 uncooked CN samples, 4 were found positive for *S. aureus*. Out of 10 OC CN samples from brand-1, two were positive for *S. aureus. *In the case of brand 2, two out of 10 raw CN samples were found positive for *S. aureus* which was confirmed by cultural examination, biochemical test, and PCR. A summary of the isolation of *S. aureus* and other Staphylococci is presented in Figure 2.

### Gram stain characteristics

In Gram stain, the organism showed violet in color, Gram-positive, and cocci in shape and arranged in clusters resembling grapes under a light microscope; these characteristics were identical to those mentioned by Ema et al. [37].

### Biochemical profile

In the coagulase test, *S. aureus* converts rabbit fibrinogen to fibrin and clotting occurs. Out of 60 samples, 8 samples showed a positive coagulase test, and 52 samples were found negative in the coagulase test. All the tested isolates fermented dextrose, maltose, lactose, sucrose, and mannitol and produced only acid. Acid production was indicated by the color change from reddish to yellowish. All isolates were positive in catalase and MR-VP tests and were found negative in the indole production test. 

### Molecular detection of S. aureus by PCR

**Figure 3.**
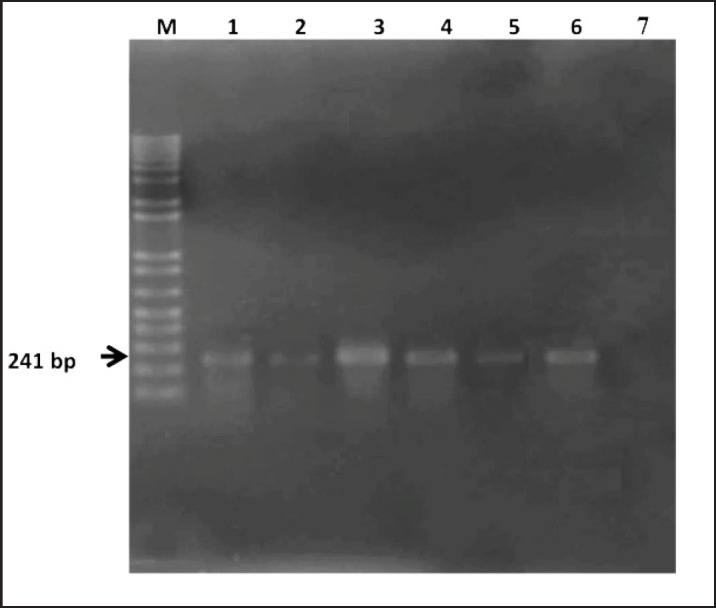
Identification of *Staphylococcus *spp. by amplification of *16S rRNA* gene by PCR. Lane M: 1kb DNA ladder; Lane 1-5: DNA of bacteria isolated from chicken nugget; Lane 6: Positive control; Lane 7: Negative control.

**Figure 4.**
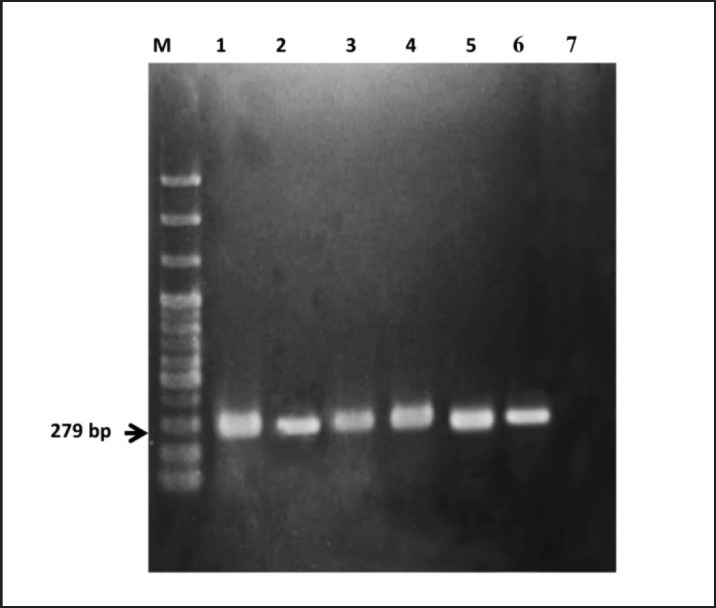
Identification of *S. aureus* by amplification of *nuc* gene by PCR. Lane M: 1kb DNA ladder; Lane 1–5: DNA of bacteria isolated from chicken nugget; Lane 6: Positive control; Lane 7: Negative control.

The identification of *Staphylococcus* spp. and *S. aureus* was validated molecularly by the *16S rRNA* and *nuc* gene-based PCR technique by amplifying 241 bp and 279 bp fragments of DNA. The results of PCR are displayed in Figures 3 and 4. These results concur with those of Khandoker et al. [35].

### PCR by sea gene-specific primer

None of the isolates were found positive for the *sea* gene suggesting that they do not produce enterotoxin (picture not shown).

### Results of antibiotic sensitivity assay

**Figure 5.**
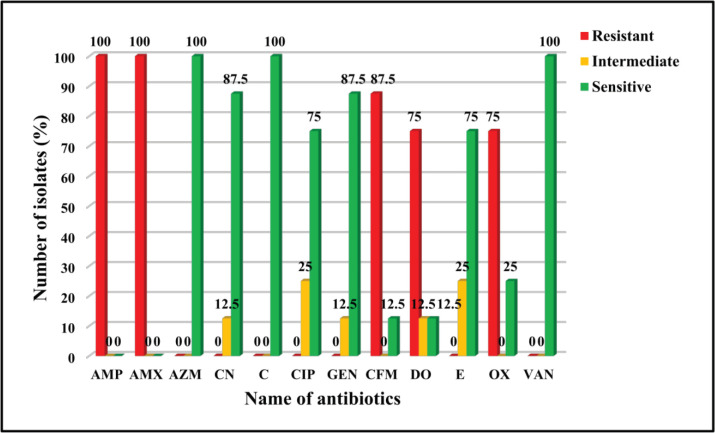
Summary of antibiogram profile of *S. aureus *against 12 antibiotics. *Staphylococcus aureus* were resistant to Ampicillin (100%), Amoxicillin (100%), Cefixime (87.5%), Doxycycline (75%), and Oxacillin (75%); intermediately sensitive to Cephalexin (12.5%), Ciprofloxacin (25%), Gentamicin (12.5%), Doxycycline (12.5%), and Erythromycin (25%); and sensitive to Azithromycin (100%), Cephalexin (87.5%), Chloramphenicol (100%), Ciprofloxacin (75%), Gentamicin (87.5%), Cefixime (12.5%), Doxycycline (12.5%), Erythromycin (75%), Oxacillin (25%), and Vancomycin (100%).

Eight isolates of *S. aureus *were tested against 12 antibiotics using disk diffusion methods. Among them, Ampicillin (100%) and Amoxicillin (100%) showed the highest resistant pattern followed by Cefixime (87.5%), Oxacillin (75%), and Doxycycline (75%). The highest sensitivity pattern was observed for Azithromycin (100%), Vancomycin (100%), and Chloramphenicol (100%) followed by Cephalexin (87.5%), Gentamicin (87.5%), Erythromycin (75%), Ciprofloxacin (75%), Oxacillin (25%), Cefixime (12.5%), and Doxycycline (12.5%), and intermediately sensitive to Erythromycin (25%), Ciprofloxacin (25%), Cephalexin (12.5%), Gentamicin (12.5%) and Doxycycline (12.5%). The results of the antibiotic sensitivity assay are shown in (Table 2 and Fig. 5). 

These study findings agree with the previous study conducted by Ema et al. [37], Sultana et al. [38], Martins et al. [39], Singh et al. [40], Tagoe et al. [41], Pyzik and Marek [42], Sharma and Mazumdar [43], and Pekana and Green [44]. They found the highest resistance rate for Amoxicillin, Ampicillin, and Tetracycline against *S. aureus*. Sabuj et al. [32] reported that all the isolated *Staphylococcus* spp. from street-vended fast foods in Mymensingh demonstrated sensitivity to Ciprofloxacin, Gentamycin, Azithromycin, and Chloramphenicol which is identical to our present research findings. *Staphylococcus *spp. was reported as Vancomycin-resistant by Kalantari et al. [45], Yurdakul et al. [46], Tabashsum et al. [47], Eromo et al. [48]; in this study, the *Staphylococcus* was found sensitive to Vancomycin. One possible explanation is that Vancomycin is not widely used in Bangladesh. Parvin et al. [49] reported a significant incidence of MDR MRSA in frozen chicken flesh samples. The findings show that CN marketed in local markets is contaminated with MDR *S. aureus*, which could be a risk to public health and a potential cause of resistant Staphylococcal food-borne poisoning.

**Table 2.** Antibiogram profile of *S. aureus.*

Antimicrobial class	Antimicrobial agents	No. of isolates (%)		
		Resistant	Intermediate	Sensitive
Penicillins	Ampicillin (AMP) Amoxicillin (AMX) Oxacillin (OX)	8 (100%)	0 (0.0%)	0 (0.0%)
		8 (100%)	0 (0.0%)	0 (0.0%)
		6 (75.0%)	0 (0.0%)	2 (25.0%)
Macrolide	Azithromycin (AZM) Erythromycin (E)	0 (0.0%)	0 (0.0%)	8 (100%)
		0 (0.0%)	2 (25.0%)	6 (75.0%)
Cephalosporin	Cefalexin (CN) Cefixime (CFM)	0 (0.0%)	1 (12.5%)	7 (87.5%)
		7 (87.5%)	0 (0.0%)	1 (12.50%)
Chloramphenicol	Chloramphenicol (C)	0 (0.0%)	0 (0.0%)	8 (100%)
Fluoroquinolone	Ciprofloxacin(CIP)	0 (0.0%)	2 (25.0%)	6 (75.0%)
Aminoglycoside	Gentamicin (GEN)	0 (0.0%)	1 (12.5%)	7 (87.5%)
Tetracyclines	Doxycycline (DO)	6 (75.0%)	1 (12.5%)	1 (12.5%)
Glycopeptide	Vancomycin(VAN)	0 (0.0%)	0 (0.0%)	8 (100%)

## Conclusion

This is the first report on the isolation and identification of S. aureus from CN in Bangladesh. GSC CN reduced the TSC more than OC and uncooked CN. *S. aureus* from CN samples is considered a major foodborne pathogen causing severe gastroenteritis due to the production of heat-resistant enterotoxin in the human intestine. In addition, this study has also found that these CN samples are contaminated with MDR *Staphylococcus* spp. This is very alarming as the pathogen represents a public health hazard. The results of this study highlight the significance of enhancing hygienic practices for both consumers and handlers and raising public knowledge of sanitation at all stages of production, handling, transportation, and processing of CN to prevent the contamination and spread of resistant bacteria and also to prevent food-borne illness. 

## List of Abbreviations

CFU, Colony forming unit; CN, Chicken nugget; GSC, Gas stove cooked; MDR, Multi drug resistant; OC, Oven cooked; PBS, Phosphate-buffer solution; PCR, polymerase chain reactions; TSC, Total staphylococcal count.
